# Editorial: Gender and wellbeing

**DOI:** 10.3389/fpsyg.2022.1080114

**Published:** 2022-11-29

**Authors:** Amelia Díaz, Maria Jose Garcia Oramas, M. Pilar Matud

**Affiliations:** ^1^Department of Personality, Psychological Assessment and Treatment, Universidad de Valencia, Valencia, Spain; ^2^Department of Health Sciences, Universidad Veracruzana, Xalapa, Mexico; ^3^Department of Clinical Psychology, Neuropsychology and Methodology, Universidad de La Laguna, San Cristóbal de La Laguna, Spain

**Keywords:** wellbeing, coping styles, gender roles, emotion, sexism, gender stereotypes

## Introduction

The selected contributions aim to improve the knowledge of gender, and gender norms as a social driver of wellbeing. Gender is an important social determinant of health and wellbeing throughout the life course, and it refers to the roles, attitudes, feelings, activities, behaviors, attributes and opportunities that any society considers appropriate for boys and girls, and for women and men. It is understood as a complex social system that defines women and men as different and structure the life experience of all people (Heise et al., 2019). Gender norms are the collective unspoken and spoken rules and expectations about how girls and boys, men and women should behave, feel, think and interact (Weber et al., 2019). These norms include descriptive norms (collective beliefs about what people do) and normative expectations (what others think they should do) that are internalized and enacted through the life course.

## Contributions

The wellbeing and desires of women about their condition and their bodies around pregnancy, childbirth and postpartum are secondary when profit and efficiency are part of the equation. In this context, Santana da Silva et al. analyzed policies and practices around pregnancy, childbirth and the postpartum phase in Brazil and France by conducting a qualitative, multicenter, international study, focusing on the female condition and the social role of women's bodies. They found that, even though there is a better policy in France than in Brazil when it comes to respect to women's autonomy, the basic guarantees of women's sexual and reproductive rights, including the right to privacy, self-determination, freedom, and individual autonomy are aspects to be improved.

Gender differences were studied in wellbeing and mental health in a diverse variety of situations. Yuan et al. investigated the effect of emotional reactivity on marital quality and the mediating role of perceived partner responsiveness in Chinese Couples. Results showed that the scores of husbands' perceived partner responsiveness and marital quality were significantly higher than those of the wives. The emotional reactivity of couples was negatively correlated with perceived partner responsiveness and marital quality, while the perceived partner responsiveness was significantly positively correlated with marital quality. In Pakistan, Shahzad et al. presented the paradox that women were infected with COVID-19 at a much lower rate than men, yet the study showed that men reported higher levels of both distress tolerance and wellbeing than women. Gender differences indicated a greater overall effect of the pandemic on women vs. men. Possible explanations of these findings were based in the fact that women presented less acceptance of negative emotions than men, whilst men showed different coping with trauma, being engaged in more active coping and problem-solving than women.

When the focus is on the coping styles, O'Rourke et al. studied gender differences in the impact of different situational coping styles in everyday life situations. Situational coping was measured by the question “How well can you cope with your momentary stress level”, over a 4-week period, with the answers being monitored with the mobile health app TrackYourStress. *Positive Thinking* and *Active Stress Coping* presented significant positive impacts on situational coping in the total sample. *Social Support* showed a significant positive effect on situational coping only in women, whereas *Active Stress Coping* had a significant positive effect on situational coping in men. These results suggest that the differences in coping styles could be more effective for women than for men in daily life.

A situation where the sexism stereotypes seem to work is in the intergenerational family narratives. Research on gender and the family narrative is limited but it seems that intergenerational knowledge of one's family history is associated with positive mental health and wellbeing, where culture and gender are critical factors in this association. Elias and Brown showed that family narratives were in line with sexist stereotypes, perpetuating ideas in which women assume the role of family caregivers and men that of family material providers. The study highlights the need for future research in the field, extending research beyond that performed on white, middle-class, heterosexual samples, and studying the influence of sexism in intergenerational family narratives and in the poorer wellbeing of women when compared with men.

In the systematic revision performed by Van Hoy and Rzeszutek the relationship between sociodemographic, - including age and gender -, intrapersonal, and work-related factors, burnout and psychological wellbeing among psychotherapists were analyzed. Burn out is considered a negative factor of wellbeing. Related to age, there is consensus in that younger psychotherapists tend to report increased levels of burnout symptoms in comparison to older and more experienced colleagues, whereas in terms of gender, there are more discrepancies in existing studies. Therefore, the authors consider that future research needs to be done focusing on the different ways men and women can experience burnout. For instance, women score higher on emotional exhaustion, whereas men score higher on depersonalization. On the other hand, as regards to quality of life, considered as a positive factor of wellbeing, there are no systematic studies in the literature thus far reporting on the issue among psychotherapists. Therefore, future research on this topic is also needed.

Although the literature revised (Ryan et al., 1999; Dittmar et al., 2014) presented the moderating role of gender between materialism values and subjective wellbeing, not all studies could confirm it. This is the case of Zhou et al. which determined the cited relationship through a meta-analysis selecting 56 studies from 1998 to 2022, taking moderators variables, among others, gender and age, found that only age presented a moderating effect in the way that the higher the mean age, the weaker the negative correlations between materialism values and subjective wellbeing.

## Conclusion

The diversity of contributions, using different theoretical and methodological approaches, show the complexity in the relationship between gender and wellbeing. In addition, the contributors all agree in that more research needs to be done on the topic to understand how gender as a social determinant impacts on health and wellbeing.

## Author contributions

The manuscript has been written and revised equally by AD, MO, and MM. All authors contributed to the article and approved the submitted version.

## Conflict of interest

The authors declare that the research was conducted in the absence of any commercial or financial relationships that could be construed as a potential conflict of interest.

## Publisher's note

All claims expressed in this article are solely those of the authors and do not necessarily represent those of their affiliated organizations, or those of the publisher, the editors and the reviewers. Any product that may be evaluated in this article, or claim that may be made by its manufacturer, is not guaranteed or endorsed by the publisher.

## References

[B1] Dittmar H. Bond R. Hurst M. Kasser T. (2014). The relationship between materialism and personal wellbeing: A meta-analysis. J. Pers. Soc. Psychol. 107, 879–924. 10.1037/a0037409 25347131

[B2] Heise L. Greene M. E. Opper N. Stavropoulou M. Harper C. Nascimento M.. (2019). Gender inequality and restrictive gender norms: framing the challenges to health. Lancet 393, 2440–2454. 10.1016/S0140-6736(19)30652-X 31155275

[B3] Ryan R. M. Chirkov V. I. Little T. D. Sheldon K. M. Timoshina E. Deci E. L. (1999). The American dream in Russia: Extrinsic aspirations and wellbeing in two cultures. Pers. Soc. Psychol. Bull. 25, 1509–1524. 10.1177/01461672992510007

[B4] Weber A. M. Cislaghi B. Meausoone V. Abdalla S. Mejía-Guevara I. Loftus P.. (2019). Gender norms and health: insights from global survey data. Lancet 393, 2455–2468. 10.1016/s0140-6736(19)30765-2 31155273

